# Multiple simultaneous embolic cerebral infarctions 11 months after COVID-19

**DOI:** 10.1186/s12959-021-00304-8

**Published:** 2021-08-18

**Authors:** Rajiv Advani, Torbjørn Austveg Strømsnes, Espen Stjernstrøm, Sebastian T. Lugg

**Affiliations:** 1 Stroke Unit, Department of Neurology, Oslo University Hospital, Oslo, Norway; 2 Neuroscience Research Group, Stavanger University Hospital, Stavanger, Norway; 3 Department of Clinical Medicine, University of Bergen, Bergen, Norway; 4 Department of Radiology, Oslo University Hospital, Oslo, Norway; 5 Institute of Inflammation and Ageing, University of Birmingham, Birmingham, UK

**Keywords:** COVID-19, Pulmonary Embolus, Stroke, Thrombolysis, Anticoagulation, Coagulopathy

## Abstract

**Background:**

The coronavirus disease (COVID-19) pandemic has led to an unprecedented worldwide burden of disease. However, little is known of the longer-term implications and consequences of COVID-19. One of these may be a COVID-19 associated coagulopathy that can present as a venous thromboembolism (VTE) and further, as multiple paradoxical cerebral emboli.

**Case presentation:**

A 51 year old man presented to the emergency department with multiple simultaneous embolic cerebral infarctions 11 months after mild COVID-19. In the subacute phase of the COVID-19 illness the patient developed increasing shortness of breath and was found to have an elevated D-dimer and multiple bilateral segmental pulmonary emboli. He was subsequently treated with 3 months of anticoagulation for a provoked VTE. The patient then presented 11 months after the initial COVID-19 diagnosis with multiple simultaneous cerebral infarctions where no traditional underlying stroke etiology was determined. A patent foramen ovale (PFO) and an elevated D-dimer were found suggesting a paradoxical thromboembolic event due to an underlying coagulopathy.

**Conclusions:**

This case report highlights the one of the potentially more serious complications of long-term COVID-19 where VTE due to a persistent coagulopathy is seen almost a year after the initial illness. Due to the highly prevalent nature of PFO in the general population, VTE due to COVID-19 associated coagulopathy could lead to ischemic stroke. This case report highlights the possibility for an underlying COVID-19 associated coagulopathy which may persist for many months and beyond the initial illness.

## Background

The longer-term implications and consequences of COVID-19 remain largely unmapped. One of the previously identified longer-term complications is a COVID-19 associated coagulopathy. This associated coagulopathy can present as a venous thromboembolism (VTE) and, as yet unreported, multiple paradoxical cerebral emboli. Furthermore, the temporal correlation of the emergence of a COVID-19 associated coagulopathy to the primary illness remains unknown. In cases of milder COVID-19 symptoms, an associated coagulopathy can remain undiagnosed and result in thromboembolic events after the initial illness.

## Case presentation

A 51 year old man presented to the Emergency Department (ED) with acute onset left sided peripheral facial palsy and a complete right sided homonymous hemianopsia.

The patient was a Caucasian male of normal BMI with no prior history of hypertension, hypercholesterolemia, diabetes, smoking and or heart disease. He had been in good health until March 2020 when he returned from a group holiday to Austria. The patient started feeling unwell with a fever and was diagnosed with coronavirus disease (COVID-19) along with several members of the group that he had travelled with. A nasopharyngeal PCR swab confirmed the presence of SARS-CoV-2 RNA on the 22nd of March 2020. The patient was admitted the hospital on the 29th of March 2020 after increasing shortness of breath and onset of diarrhea and vomiting. The patient was febrile but had no oxygen requirement and was therefore diagnosed with mild COVID-19. He was admitted for observation, managed conservatively, and was discharged home 3 days later on the 1st of April 2020 after showing clinical improvement.

**Fig. 1:**
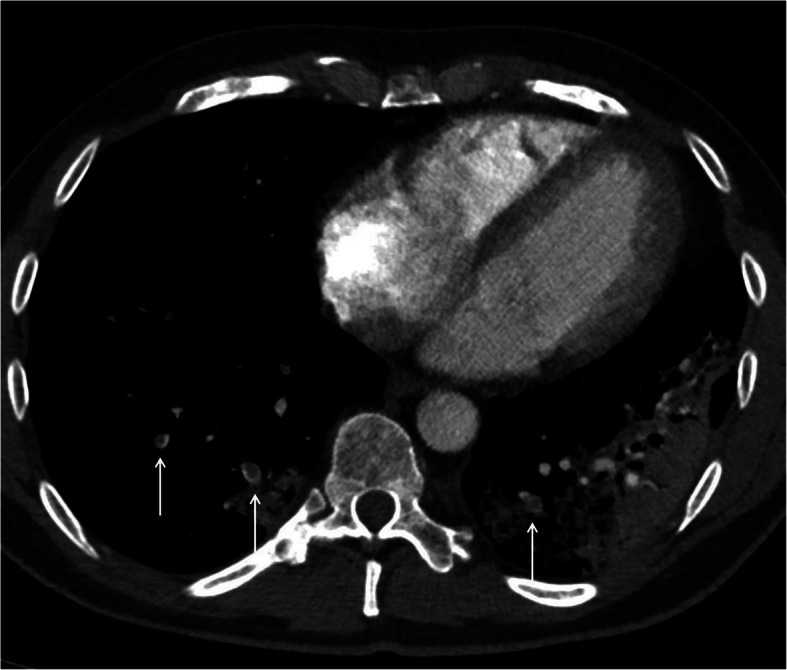
CTPA showing multiple bilateral segmental pulmonary emboli

**Fig. 2:**
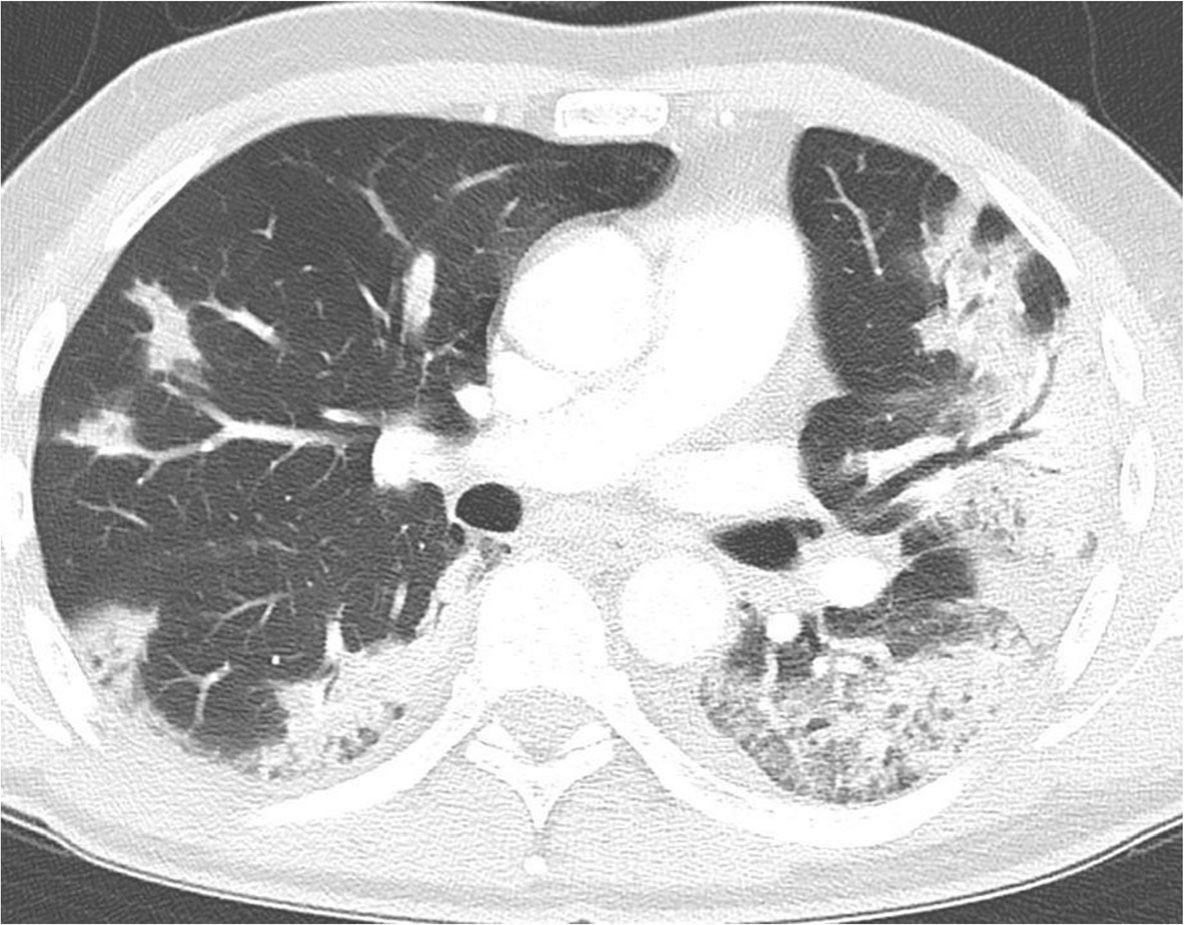
Chest CT showing bilateral COVID-19 changes

The patient was readmitted 2 days later on the 3rd of April 2020 with increasing shortness of breath. Clinically, pulmonary embolism (PE) was suspected with no signs of hemodynamic compromise. The diagnosis was confirmed by a CT Pulmonary Angiography (CTPA) (Fig. 1) which demonstrated multiple bilateral subsegmental PE with no signs of right sided heart strain. The CTPA also demonstrated bilateral ground glass and consolidation in the lungs in keeping with COVID-19 infection (Fig. 2). The CT examination was performed using the Siemens® Somatom Definition Flash using GE Healthcare® Omnipaque intravenous contrast and a standardized CTPA protocol. The patient had a D-dimer > 4.0 mg/L (normal range 0.2–0.6 mg/L). The Chest CT performed during the CTPA also shows the radiological extent of the COVID-19 illness in the lungs (Fig. 2). The patient was started on Low Molecular Weight Heparin (LMWH) with bridging to direct oral anticoagulation (DOAC) on a dose of 20 mg Rivaroxaban OD. The patient continued DOAC treatment for a total of three months until the beginning of July 2020.

On the 26th of February 2021, approximately 11 months after being diagnosed with COVID-19, the patient contacted the emergency medical services due to acute onset visual field disturbances. The ambulance arrived on scene minutes after the onset of symptoms. A preliminary examination by the paramedics revealed as right sided visual field defect and a left sided facial palsy. The patient had a heart rate of 70 beats per minute and blood pressure of 147/89 mm Hg. The prehospital oxygen saturation was 98 % whilst breathing room air. Based on their initial findings, the paramedics suspected a stroke, and the patient was blue lighted to the ED.

On admission to the ED the patient was taken directly to the CT lab. The patient had a temperature of 35.5 ℃, ECG showing normal sinus rhythm, blood glucose of 5.8 mmol/L, a blood pressure of 157/99 mmHg and an oxygen saturation of 99 % on room air. A rapid neurological examination revealed a left sided peripheral facial palsy, right sided homonymous upper quadrantopia. A National Institutes of Health Stroke Scale (NIHSS) exam revealed a score of 3.

A CT scan was performed including a pre- and intra- cerebral angiography. A subsequent cerebral perfusion scan was also performed. The CT examination was performed using the Siemens® Somatom Definition Flash using GE Healthcare® Omnipaque intravenous contrast and standardized protocols for angiography and perfusion.

**Fig. 3:**
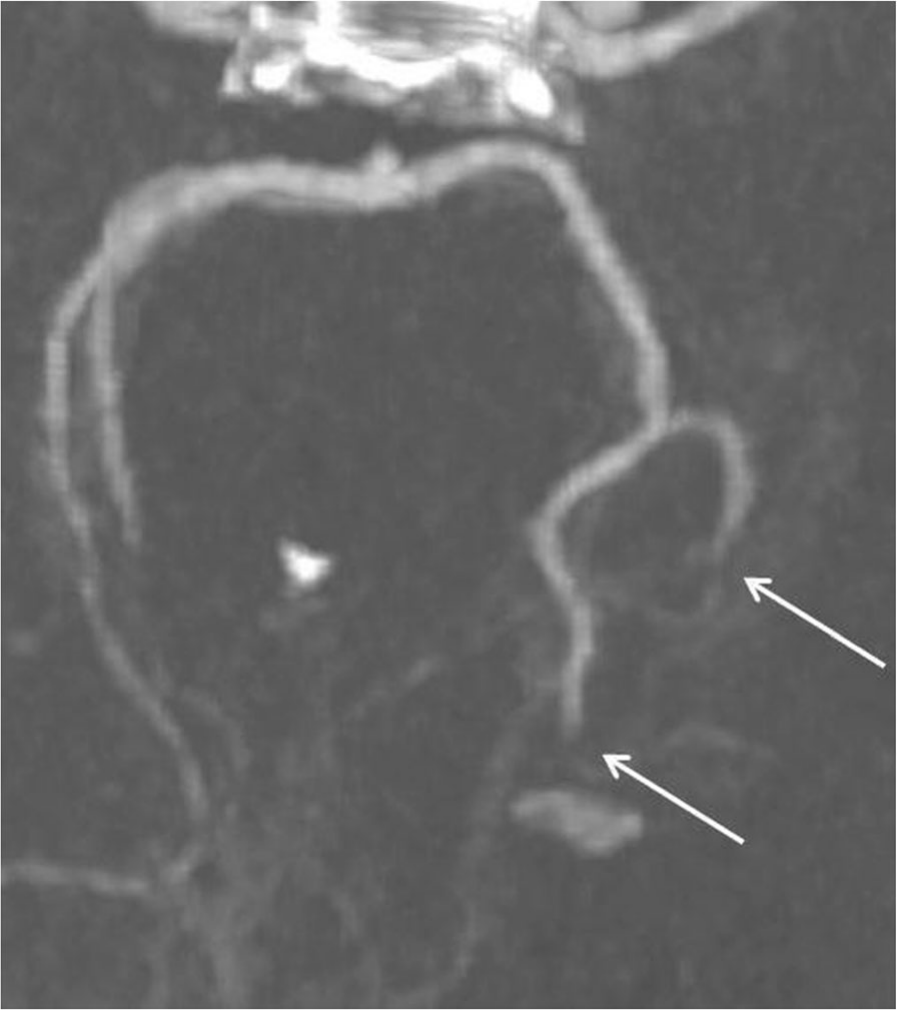
CTA showing occlusions of the P2 and P3 segments of the left posterior cerebral artery

**Fig. 4:**
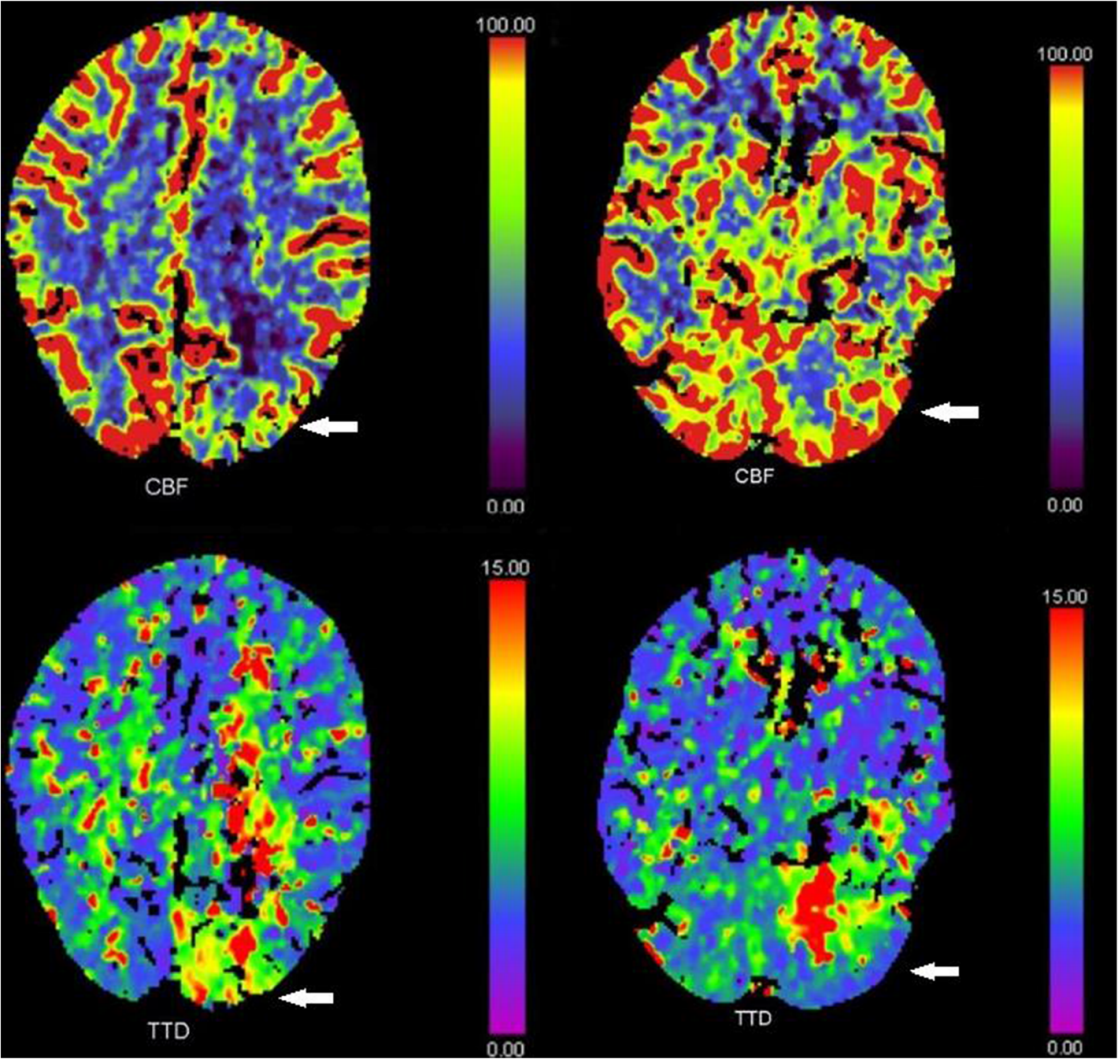
CTP showing reduced CBF and increased TTD in P2 and P3 segments of the left posterior cerebral artery and the left superior cerebellar artery

The plain CT showed no signs of hemorrhage and based on the clinical suspicion of stroke the decision to treat with intravenous thrombolysis was made. Alteplase was administered at a dose of 0.9 mg/kg as per guidelines. The CT angiography three occluded intracerebral vessels, occlusions of the P2 and P3 segments of the left posterior cerebral artery (Fig. 3) and an occlusion of the left superior cerebellar artery. The CT perfusion scan show increased time to drain (TTD) and reduced cerebral blood flow (CBF) in the afore mentioned occluded vascular territories (Fig. 4).

The patient was admitted to the stroke unit and showed neurological improvement after Alteplase administration. The following day the patient had a NIHSS of 0.

**Fig. 5:**
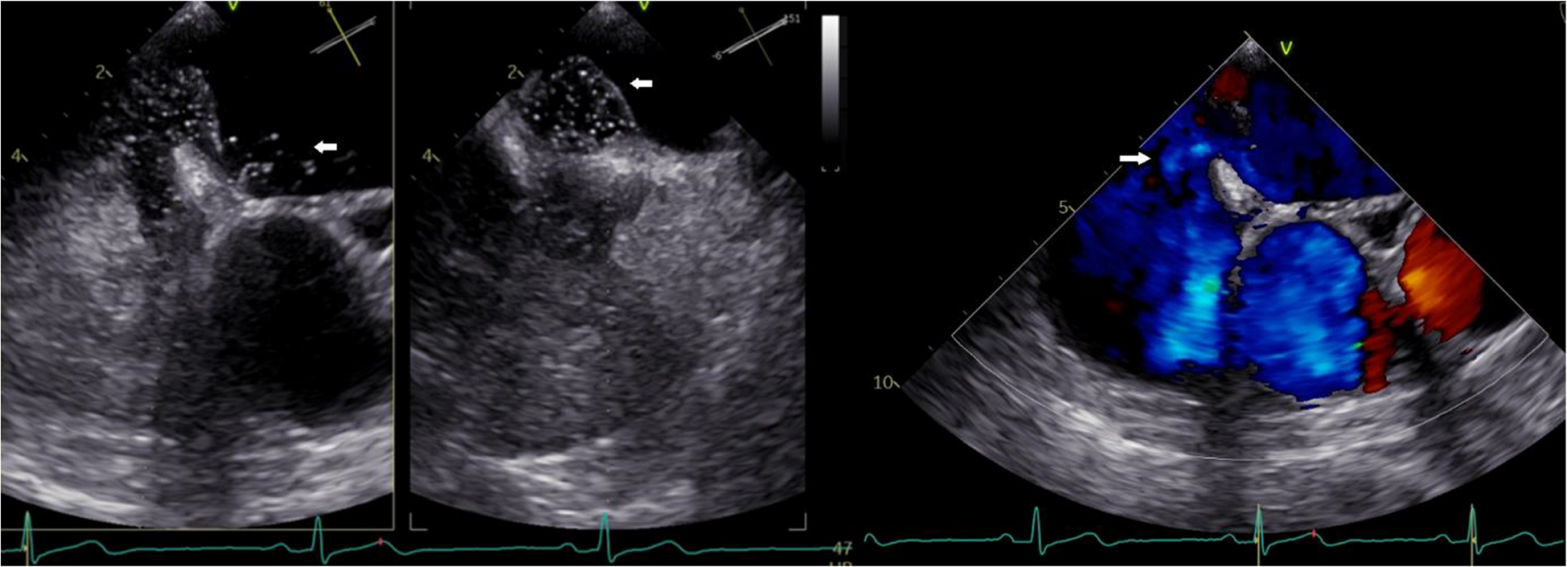
Echocardiography showing a PFO using agitated sodium chloride contrast (left) and on colour doppler (right)

During hospital admission examinations were performed to determine ischemic stroke etiology. Ultrasound of the pre- and intra- cerebral vessels showed no significant stenoses or occlusions. Five day in-hospital cardiac rhythm monitoring showed sinus rhythm without any signs of atrial fibrillation and or other arrythmia. The patient underwent a Trans- Thoracic Echocardiography (TTE) which was normal and a Trans- Esophageal Echocardiography (TEE) where a patent foramen ovale was detected (PFO). The right to left shunting was visualized on color doppler and whilst using contrast (Fig. 5). A blood work up during admission showed a normal ESR, normal levels of Immunoglobulin G and M, normal levels of Protein C and S and normal Factor Xa activity. Immunological assays did not detect the presence of rheumatoid factor, ANCA, ANA, anti-CCP antibodies. Furthermore, no factor V Leiden mutation was detected nor the presence of Lupus anticoagulant. The only abnormal finding was an elevated D-dimer of 1.2 mg/L (normal range 0.2–0.6 mg/L). A full body CT was performed including a CTPA and a CT venography of the lower extremities. The full body CT showed no signs of malignancy. The CTPA showed no evidence of pulmonary embolus and the CT venography of the lower extremities ruled out deep vein thrombosis (DVT).

The patient was discharged with a NIHSS of 0 following an Embolic Stroke of Unknown Source (ESUS). The patient is currently being treated with 20 mg Rivaroxaban OD, and atranscatheter closure of his PFO has been scheduled.

## Discussion and conclusions

Patients with COVID-19 are frequently found to have underlying coagulopathy. This is demonstrated by evidence of microthrombosis/venous thrombus, elevated D-dimer and fibrinogen, activated complement system and von Willebrand Factor (vWF), increased inflammatory cytokines (IL-1β, IL-6) and antiphospholipid antibodies, as well as macrophage/endothelial cell dysfunction [1]. The resultant COVID-19 associated coagulopathy most commonly complicates in the form of venous thromboembolic event (VTE) such as PE or deep vein thrombosis. However, arterial thromboembolism in the form of acute ischemic stroke or acute coronary syndrome more rarely can occur.

Our case presented with VTE in the form of PE during the sub-acute phase of COVID-19 infection, then later, arterial thromboembolism in the form of acute ischemic stroke 11 months after COVID-19.

Large arterial vessel stroke can be a presenting feature of COVID-19 in young patients. This was demonstrated in early case series during the pandemic, of which the majority (4 of 5) had elevated levels of D-dimer at diagnosis [2]. There are however no published case reports to date of acute stroke as a presentation of suspected paradoxical thromboembolic recurrence in a patient with previous COVID-19 infection. As part of an extensive work up for the acute stroke the patient was found to have coagulopathy in the form of elevated D-dimer. Studies have previously shown D-dimer to be elevated in a sub-set of COVID-19 patients at follow up [3]. Up to 25 % of patients have shown to have elevated D-dimer levels 4 months after the initial infection [4], though the clinical significance of this remains unclear.

Of interest PFO was also found, which has previously been reported to complicate PE in the context of COVID-19 infection in a young female [5]. Underlying PFO has also been found in a young male with COVID-19 presenting with acute right cerebellar infarct [6]. Tough we are not certain, we suspect that the underlying long-term COVID-19 associated coagulopathy in the setting of a PFO led to paradoxical multiple simultaneous embolic cerebral infarctions in this young patient, with no other cause found after extensive investigations.

Venous thromboprophylaxis is now routinely considered for patients admitted with COVID-19 infection. There is little evidence to suggest that COVID-19 infection in the absence of clinically evident VTE warrants the use of therapeutic anticoagulation to address an underlying coagulopathy. There are similar short-term risks of both thrombosis and bleeding in these patients [7]. In the context of VTE, as in the early stages of this case, the patient was treated with a DOAC for 3 months which is a standard treatment duration for a provoked VTE [8]. There is however almost no evidence at present to suggest that these patients should be treated with DOAC over a prolonged period of time. Up to 35 % of the general population have a PFO. This combined with the unknown extent of COVID-19 related coagulopathy; a major challenge looms for stroke physicians worldwide [9].

This case reports highlights the more serious, longer term complications of COVID-19 where multiple embolic infarctions presented almost 1 year after the acute illness. The COVID-19 associated coagulopathy potentially persisting many months after diagnosis in patients with a milder clinical course of illness.

The long-term consequences of COVID-19 infection are only just beginning to surface potentially impacting a great number of individuals. More studies and reports are needed to map out the potential long-term consequences of COVID-19 associated coagulopathy.

## Data Availability

The supporting data is kept in the patient medical journal and radiological systems at Oslo University Hospital.

## Abbreviations

ED: Emergency Department
BMI: Body mass index
Covid-19: Coronavirus disease, *sars-CoV-2*
*PCR*: *Polymearase chain reaction*
PE: Pulmonary embolism
*VTE*: *Venous thromboembolism*
PFO: Patent foramen ovale
DVT: Deep vein Thrombosis
CT: Computer tomography
CTPA: CT pulmonary angiography
LMWH: Low Molecular Weight Heparin
DOAC: Direct oral anticoagulation
ECG: Electrocardiography
NIHSS |: National Institutes of Health Stroke Scale
TTD: Time to drain
CBF: Cerebral blood flow
ESR: Erythrocyte sedimentation rate
TEE: Trans- Esophageal Echocardiography
ANA: Anti nuclear antibodies
ANCA: Anti-neutrophil cytoplasmic antibodies
Anti-CCP: Anti- cyclic citrullinated peptides (CCP)
ESUS: Embolic Stroke of Unknown Origin
